# Assessment of the Relaxation-Enhancing Properties of a Nitroxide-Based Contrast Agent TEEPO-Glc with *In Vivo* Magnetic Resonance Imaging

**DOI:** 10.1155/2019/5629597

**Published:** 2019-12-21

**Authors:** Maiju Soikkeli, Mikko I. Kettunen, Riikka Nivajärvi, Venla Olsson, Seppo Rönkkö, Johanna P. Laakkonen, Vesa-Pekka Lehto, Jari Kavakka, Sami Heikkinen

**Affiliations:** ^1^Department of Chemistry, University of Helsinki, FI-00014 Helsinki, Finland; ^2^A. I. Virtanen Institute for Molecular Sciences, University of Eastern Finland, FI-70211 Kuopio, Finland; ^3^Department of Applied Physics, University of Eastern Finland, FI-70211 Kuopio, Finland

## Abstract

Magnetic resonance imaging examinations are frequently carried out using contrast agents to improve the image quality. Practically all clinically used contrast agents are based on paramagnetic metals and lack in selectivity and specificity. A group of stable organic radicals, nitroxides, has raised interest as new metal-free contrast agents for MRI. Their structures can easily be modified to incorporate different functionalities. In the present study, a stable nitroxide TEEPO (2,2,6,6-tetraethylpiperidin-1-oxyl) was linked to a glucose moiety (Glc) to construct a water-soluble, potentially tumor-targeting compound with contrast-enhancing ability. The ability was assessed with *in vivo* MRI experiments. The constructed TEEPO-Glc agent proved to shorten the *T*_1_ relaxation time in tumor, while the *T*_1_ time in healthy brain tissue remained the same. The results indicate the potential of TEEPO-Glc as a valuable addition to the growing field of metal-free contrast enhancement in MRI-based diagnostics.

## 1. Introduction

Magnetic resonance imaging (MRI) is one of the most prominent imaging modalities due to its superior versatility, soft tissue contrast, and resolution. Although optimizing imaging conditions often leads to excellent image quality, in some cases, the contrast between pathologies and healthy tissue is improved by utilizing contrast agents (CAs). Contrast agents can also increase the signal-to-noise ratio (SNR) leading to better image quality and resolution. Traditional contrast agents are mainly based on a paramagnetic gadolinium metal due to its seven unpaired electrons, high magnetic moment, and long electron spin relaxation [1, 2]. However, free Gd^3+^ is toxic in human body mainly due to its identical ionic size with Ca^2+^. As a result, it can potentially bind to Ca^2+^ channels and disturb protein synthesis [3]. Therefore, gadolinium is tightly bound with organic ligands, increasing both its kinetic inertness and thermodynamic stability [2]. In 2006, it was reported that the use of gadolinium-based contrast agents (GBCAs) on patients with renal impairment could cause nephrogenic systemic fibrosis (NSF) [4]. Later on, the prevalence of NSF was linked with the use of less stable, linear contrast agents. With thorough patient screening and restricted use of certain GBCAs, their use has been generally considered safe. Until 2015, it was prevailed that with patients going through several scans with GBCAs, gadolinium can deposit in brain [5]. Although the retention of gadolinium has not been found to be harmful for patients, the European Medical Agency (EMA) recommended suspension or restricted the use of four linear GBCAs [6]. Another concerning aspect, due to the broad use of GBCAs, is the findings of anthropogenic gadolinium in aquatic environments [7] and even in drinking water [8]. Therefore, the development of new, metal-free contrast agents has accelerated.

Two general approaches to avoid the use of metals in CAs are the exploitation of chemical exchange saturation transfer (CEST) [9, 10] or hyperpolarized [11–13] MRI contrast agents. However, these methods are quite complex and often require special techniques or dedicated hardware, which often causes some restrictions to their clinical applicability. Lately, a group of contrast agents based on paramagnetic stable radicals, nitroxides, has emerged. The applicability of nitroxides in MRI was discovered already in the 1980s [14]. Nitroxide radicals have a wide range of applications in organic synthesis [15, 16], radical polymerization [17], spin labelling [18], as molecular magnets [19], and organic batteries [20–22]. Nitroxides are often prone to reduction by various natural reductants such as ascorbic acid or enzymes, which leads to formation of their diamagnetic equivalents, hydroxylamines. However, nitroxides with bulky side groups have shown remarkable stability in conditions mimicking biological matrix [23, 24]. The research of nitroxide-based contrast agents comprises both macromolecular [25–31] and small molecule [32–36] systems. It is often considered that the advantage of using polymers or nanomaterials as a backbone for nitroxides is the possibility to attach several radical centers to the contrast agent molecule. Also, large molecules often have long rotational correlation times indicating their tumbling rates, which increases the relaxivity of the contrast agent [25]. However, increasing the size of the contrast agent molecule also has unfavorable effects on tissue penetration and delivery [2].

**Figure 1:**
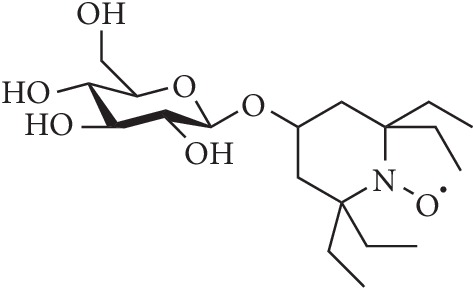
The structure of the contrast agent TEEPO-Glc.

Our study has focused on attaching a highly stable nitroxide, TEEPO (2,2,6,6-tetraethylpiperidin-1-oxyl), to natural compound moieties that can potentially act as targeting units [35, 36]. The method is commonly applied in the use of radiopharmaceuticals as tracers in PET (positron emission tomography) imaging [37]. In our first study, we presented the synthesis of TEEPO-Glc (Figure 1), where TEEPO is covalently attached to a glucose molecule [35]. One of the main advances of using glucose is its ability to increase the water solubility of the otherwise lipophilic TEEPO. Our study presented superior stability of the compound in a matrix mimicking biological environment and against a natural reductant ascorbic acid. It also displayed a preliminary study on its cytotoxicity and relaxation enhancement properties in *in vitro* NMR and phantom MRI experiments. Herein, we present the results of a more detailed *in vitro* cytotoxicity study and also the relaxation enhancing properties with *in vivo* MRI experiments.

## 2. Materials and Methods

### 2.1. *In Vitro* Cytotoxicity Study

The preparation of the TEEPO-Glc contrast agent as well as the cell viability study with HeLa cells is described in earlier publication [35]. For the *in vitro* cytotoxicity studies, HeLa cells were cultured in a complete cell culture medium composed of Dulbecco's modified Eagle's medium (DMEM, Sigma-Aldrich, UK), 10% heat-inactivated fetal bovine serum (Gibco, Life Technologies/Thermo Fisher Scientific, US), penicillin (100 U/ml), and streptomycin (100 *μ*g/ml) (Gibco, Life Technologies/Thermo Fisher Scientific, US). The cells were maintained in standard cell culture conditions (37°C, 5% CO_2_ and 95% humidity) in a Sanyo MCO-18AIC CO_2_ incubator (Sanyo Electric, Osaka, Japan). The HUVECs (human umbilical vein endothelial cells; Lonza) were grown on 0.1% gelatin-coated 100 mm cell culture dishes and passage numbers from P8 to P9 were used for the experiments. The cells were maintained in a complete medium containing M199 (Gibco, Life Technologies/Thermo Fisher Scientific, US), 15% fetal bovine serum, heparin (5 units/ml) (Sigma-Aldrich, UK), endothelial cell growth factor (20 *μ*g/ml) (ECGF, Roche Biomolecules, Switzerland), 1% L-glutamine, 1% streptomycin, and 1% penicillin, in a 5% CO_2_ incubator at 37°C.

To study the cell viability with HUVECs, the cells (10,000 cells per well) were seeded on 0.1% gelatin-coated white 96-well tissue culture plates, and they were allowed to attach for 24 h. After two washes with phosphate-buffered saline (PBS) (200 *μ*l per well), the cells were treated with TEEPO-Glc (0.2, 1, and 10 mM in culture medium) for 1, 6, and 24 h. The cell viability was measured using the CellTiter-Glo® reagent (Promega, US) with a Fluoroskan Ascent FL (Thermo Labsystems, US) luminometer according to the manufacturer's instructions. For the LDH release assay, HeLa cells (10,000 cells per well) were seeded on 96-well tissue culture plates (Corning Inc., Corning, NY, US) and HUVECs (10,000 cells per well) on 0.1% gelatin-coated 96-well tissue culture plates. They were allowed to attach for 24 h. After two washes with PBS (200 *μ*l per well), cells were treated with TEEPO-Glc (0.2, 1, and 10 mM in culture medium) for 1, 6, and 24 h. After exposure, the release of LDH was monitored from an aliquot of 50 *μ*l of the supernatant using CytoTox96® Nonradioactive Cytotoxicity assay (Promega, US) according to the manufacturer's instructions. The absorbance was determined with a Bio-Rad microplate reader (model-550) (Bio-Rad, US) at a wavelength of 490 nm. The release of LDH in untreated cells was used as a control. The cells lysed with the lysis solution provided in the LDH assay kit were used as a positive control and set at 100% LDH release. Statistical analysis was performed by Kruskal–Wallis with Dunnett's test. Differences were considered significant when *p* < 0.05.

### 2.2. *In Vivo* Experiments

C6 glioma cells (ECACC/Sigma-Aldrich, UK) were grown in a 10 cm Petri dish in 10 ml of high-glucose Dulbecco's modified eagle medium (DMEM, Sigma-Aldrich, UK) supplemented with 10% fetal bovine serum and 1% penicillin-streptomycin at 37°C in the presence of 5% CO_2_. Upon reaching approximately 80% confluence, the cells were washed twice with PBS and trypsinised with 1 ml of 0.4% trypsin solution.

All animal experiments were approved by the Animal Health Welfare and Ethics Committee of University of Eastern Finland. Female Wistar rats (*n*=11, 190–380 g, Envigo, UK) were anesthetized with intraperitoneal (i.p.) injection of ketamine 60 mg/kg (Ketalar vet 50 mg/ml, Pfizer, US) and medetomidine hydrochloride 0.4 mg/kg (Domitor vet 1 mg/ml, Orion Pharma Animal Health, Finland). C6 cells (1 × 10^6^ C6 cells per 10 *μ*l of ice-cold PBS) were implanted to stereotactic coordinates of 1 mm caudal from bregma, 2 mm to the right of the sagittal suture, and 2 mm below the top of bregma through a burr hole. Animals received postoperation pain medication (Norocarp, Vet Medic Pharmaceuticals Oy, Finland) after surgery.

MRI experiments were performed using a 9.4 T horizontal magnet interfaced to Agilent (Santa Clara, US) imaging console and a volume coil transmitter/4-channel surface coil receiver pair (Rapid Biomedical, Rimpar, Germany) on days 7–14 postsurgery. During the experiments, the animals were anesthetized with isoflurane (5% induction, 1-2% upkeep, 70 : 30 N_2_ : O_2_ gas mixture at 2 L/min) and placed inside a holder with breath monitoring (60–80 breaths per minute) and temperature control (37°C) using warm water. Axial multislice MRI data covering the tumor and normal brain were first collected. *T*_1_ (inversion-recovery FLASH (Fast Low Angle SHot), 12 inversion times between 5 and 5500 ms, 10 s delay between inversions, TR within FLASH 7.8 ms, TE 3.9 ms, 10° flip angle, 32 × 32 mm^2^ FOV, 128 × 64 data matrix, and twelve 1 mm slices) and *T*_2_ (multi spin-echo with 16 echoes collected between 8.1 and 129.8 ms, TR 2 s, 32 × 32 mm^2^ FOV, 128 × 64 data matrix, and eight 1 mm slices) maps were collected before CA injection and for up to one hour after injection. Fast gradient echo multislice imaging (TR 156 ms, TE 4.5 ms, flip angle 90°, 32 × 32 mm^2^ FOV, 256 × 128 data matrix, and eight 1 mm slices) was performed during CA injection; imaging started 1 min before the start of the injection and continued for 10 mins after the injection. For TEEPO-Glc, the final concentration was 0.5 to 2.2 mmol/kg (injection volume was 6 ml/kg with sample concentration varying between 75 and 277 mM) (*n*=7). For Gd(DTPA) (gadopentetate dimeglumine), final concentration was 0.1 mmol/kg (injection volume 1 ml/kg with 100 mM sample concentration; *n*=4; one of the animals had received injection of TEEPO-Glc approximately 70 minutes before Gd(DTPA) injection. Additionally, 4-hydroxy-TEMPO (2,2,6,6-tetramethylpiperidin-1-oxyl) at concentration 1.6 mmol/kg was injected (injection volume ∼6 ml/kg, concentration 290 mM; *n*=2; one of the animals received a Gd(DTPA) injection 60 minutes later). All data were analyzed in Matlab (Mathworks, Natick, US). Parameter maps were calculated using monoexponential fits.

## 3. Results and Discussion

### 3.1. Toxicity Effects of TEEPO-Glc in HeLa Cells and HUVECs

**Figure 2:**
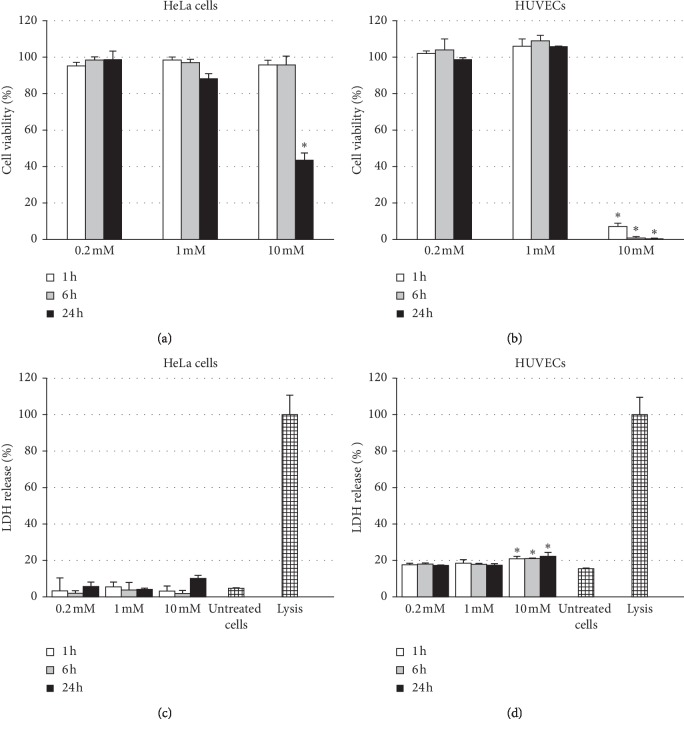
TEEPO-Glc cytotoxicity *in vitro*. Cell viability study with (a) HeLa cells (reproduced from Ref. 35 with permission from The Royal Society of Chemistry) and (b) HUVECs were determined by CellTiter-Glo® assay kit (ATP measurement), and the results (mean ± SD, *n*=4) were compared with untreated control cells whose viability was set at 100%. LDH release study with (c) HeLa cells and (d) HUVECs. Control cells were lysed with LDH assay lysis solution and set at 100% (lysis). The level of significance was set at a probability of *p* < 0.05 (^*∗*^) when compared with untreated control cells (Kruskal–Wallis with Dunnett's test).

To study the *in vitro* cytotoxicity of TEEPO-Glc, a set of cell viability and LDH release tests were performed in HeLa cells [35] and primary human endothelial cells, HUVECs. Different concentrations of TEEPO-Glc (0.2, 1, and 10 mM) were incubated for 1, 6, and 24 h. The cytotoxicity was studied using cell viability assay (Figures 2(a) and 2(b)) and LDH (lactate dehydrogenase) release assay (Figures 2(c) and 2(d)). The cell viability assay is based on quantitation of ATP (adenosine triphosphate), the amount of ATP being directly proportional to the number of living cells. The LDH release assay is a colorimetric assay for the measurement of cytoplasmic LDH enzyme activity present in all cells. LDH is released rapidly from the cytosol into culture medium upon the damage of plasma membranes of the cells.

As our earlier results showed, at a high concentration of TEEPO-Glc (10 mM) and with long incubation time (24 h), the cellular viability (*p* < 0.05) of HeLa cells decreased significantly compared with unexposed controls (Figure 2(a)). With lower concentrations (0.2 mM and 1 mM) or shorter incubation times (1 h and 6 h) TEEPO-Glc showed no effect on the cell viability. For HUVECs, TEEPO-Glc caused a significant reduction in cellular viability at a concentration of 10 mM at all detected time points, 1 h, 6 h, and 24 h (*p* < 0.05) (Figure 2(b)). However, lower concentrations of TEEPO-Glc (0.2 mM or 1 mM) did not have an effect on the cell viability. Concomitantly, results from LDH assay showed that none of the TEEPO-Glc treatments (0.2, 1, or 10 mM) caused significant membrane damage for the cultured HeLa cells (Figure 2(c)), albeit there is a slight increase with the highest concentration (10 mM) with 24 h incubation time. With HUVECs, the 10 mM concentration induced a significant LDH release (*p* < 0.05) and loss of plasma membrane integrity at all tested time points (1, 6, and 24 h) compared with untreated control cells (Figure 2(d)). Lower concentrations of TEEPO-Glc did not show any membrane-damaging effects for HUVECs at any time points tested. All in all, the TEEPO-Glc contrast agent showed toxicity only at a high 10 mM concentration and can be considered scarcely toxic in concentrations relevant to practical use.

### 3.2. Relaxation Enhancement Studies with *In Vivo* MRI

**Figure 3:**
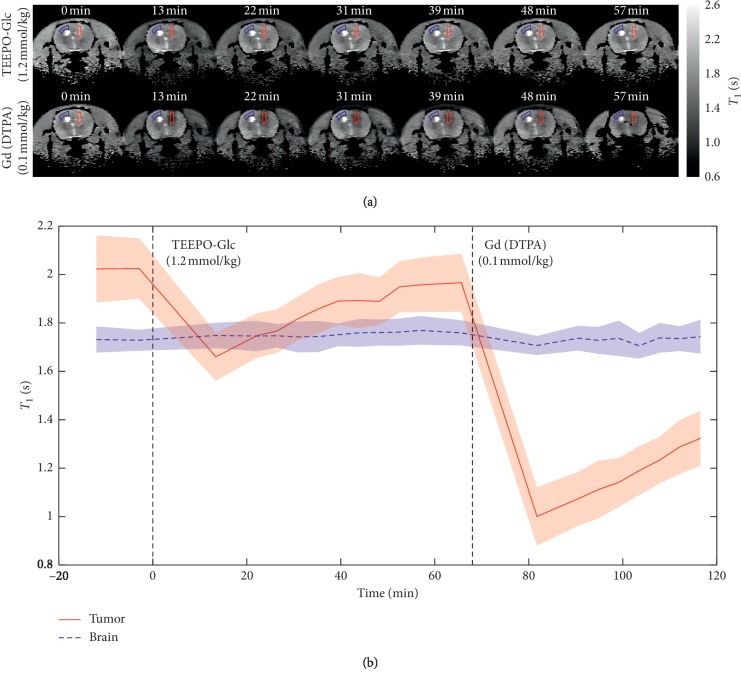
*T*_1_ mapping was performed with inversion-recovery FLASH in 9.4 T magnetic field. (a) *T*_1_ images measured before the injection (left) and approximately every 10 minutes after the injection. The red and blue areas indicate the tumor and normal brain regions of interest (ROI), respectively. (b) The corresponding *T*_1_ relaxation times in tumor and healthy brain as a function of time with error bands ( ± SD).

The relaxation-enhancing properties of TEEPO-Glc were assessed with an *in vivo* MRI study. In the study, *T*_1_ maps were collected before, during, and after contrast agent injection with an inversion-recovery FLASH imaging sequence. The maps were recorded using both TEEPO-Glc and Gd(DTPA) (gadopentetate dimeglumine) as contrast agents in order to compare the results of TEEPO-Glc to a common GBCA.  Figure 3 presents the *T*_1_ results of a representative animal.  Figure 3(a) displays the *T*_1_ images of the rat brain at approximately 10 minute intervals starting from the injection of contrast agent and *T*_1_ times with respect to the time after the injection. After 60 minutes of the TEEPO-Glc injection, the animal received the Gd(DTPA) injection. The *T*_1_ values were determined both in tumor and in normal, healthy brain. Their regions of interest (ROI) are outlined in red and blue, respectively, in Figure 3(a).  Figure 3(b) displays the average *T*_1_ relaxation times within ROI with respect to the time, and the error bands represent the standard deviation (±SD) within ROI. The differences in the preinjection *T*_1_ times between tumor and normal brain area arise from the existing contrast between healthy and malignant tissue. The results display a clear drop in the *T*_1_ relaxation time in tumor after the TEEPO-Glc injection. The effect was at strongest between 10 and 15 minutes after the injection resulting in a decrease of approximately 20% in *T*_1_. After 50 minutes, the *T*_1_ value had returned back to the level of preinjection *T*_1_ relaxation time. Judging from the high stability of TEEPO-Glc, this is most likely due to the contrast agent clearance instead of bioreduction [35]. In the healthy brain, no decrease in the *T*_1_ times was detected. Regarding the accumulation and retention time, TEEPO-Glc showed similar behaviour to Gd(DTPA) in the experiments, albeit the relaxation effect is much higher with Gd(DTPA) (Figure 3). The compared *T*_1_ decreases in tumor are also in accordance with the *r*_1_ relaxivity values determined for the compounds *in vitro*. The *r*_1_ of TEEPO-Glc was determined to be 0.13 mM^−1^ s^−1^ in 9.4 T which is similar to the values calculated from the earlier *in vitro* NMR and phantom MRI studies (0.12 mM^−1^ s^−1^ in 11.7 T and 0.23 mM^−1^ s^−1^ in 1.5 T field) [35]. The *r*_1_ value of Gd(DTPA) is significantly higher, 4 mM^−1^ s^−1^ [38], which can be observed as a stronger decrease in the *T*_1_ time after the Gd(DTPA) injection. The higher relaxivity of Gd(DTPA) is supposedly due to the fact that gadolinium has seven unpaired electrons, whereas TEEPO-Glc has one unpaired electron. Additionally, with Gd(DTPA), the relaxation enhancement is the result of both inner- and outer-sphere relaxation. Inner-sphere relaxation is caused by the water molecule directly coordinating to the paramagnetic center, and outer-sphere relaxation is a result of water molecules diffusing close to the contrast agent molecule [1, 2]. With TEEPO-Glc, only the outer-sphere relaxation is relevant as there is no direct bond between the water molecule and the nitroxide. The *T*_2_ values in tumor were not affected by TEEPO-Glc even though it showed a good contrast in the previous phantom study. Similar to GBCAs, nitroxides seem to shorten both *T*_1_ and *T*_2_ times, but the relative effect in tissue is much smaller for *T*_2_ than for *T*_1_ making them primarily *T*_1_ contrast agents [1].

Concentration of TEEPO-Glc was varied across the experiments to assess the effect of dose on the apparent CA concentration in the tumor. The relative concentrations were calculated from the relaxation rates (*R*_1_ = 1/*T*_1_) and the *r*_1_ values (0.13 mM^−1^ s^−1^ for TEEPO-Glc and 4 mM^−1^ s^−1^ for Gd(DTPA)). The apparent maximal tumor concentrations measured at ∼10 mins after injection for both compounds are shown in Table 1. Although lower apparent concentration was observed at the lowest injected dose (0.5 mmol/kg), the higher TEEPO-Glc doses all showed relatively similar apparent tumor concentrations. The concentrations have also been normalized to the injected dose (*μ*mol) to derive an approximate %ID/g value (percent of injected dose per gram of tissue) (Table 1). The apparent %ID/g were 0.19 ± 0.09 and 0.25 ± 0.09, for TEEPO-Glc and Gd(DTPA), respectively. This implies similar initial uptake of the two contrast agents despite different concentrations. The underlined animal (animal 3) received both TEEPO-Glc and Gd(DTPA) injection (see Table 1).

**Figure 4:**
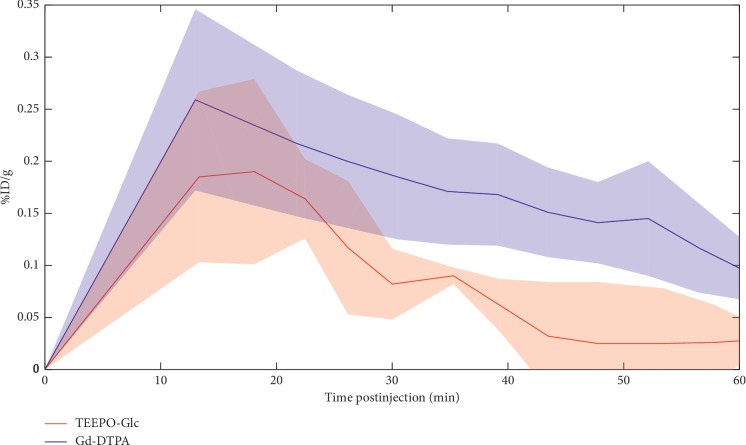
Apparent CA concentrations of TEEPO-Glc and Gd(DTPA) as a function of time postinjection. TEEPO-Glc was disappearing from tumor at a faster rate than Gd-DTPA.

The apparent contrast agent concentrations in the tumor ROI are presented with respect to postinjection time in Figure 4. The %ID/g is presented as an average of all the animals listed in Table 1. The loss of TEEPO-Glc contrast was faster than Gd(DTPA) (*p* < 0.01, Student's *t*-test, Figure 4), with a mean lifetime of 23 ± 8 min and 49 ± 10 min for TEEPO-Glc and Gd(DTPA), respectively. Together the results on apparent TEEPO-Glc concentration in tumor and the fast elimination indicate low targeting effect. Consequently, we were interested in looking into the accumulation and relaxation enhancing properties of the radical moiety without the glucose unit. However, the nitroxide radical, 4-hydroxy-TEEPO, is highly lipophilic and consequently insoluble in either pure saline or saline doped with 10% DMSO or TWEEN20/80 at desired concentrations and could not act as a reference to review the targeting effect of the glucose unit. Additionally, 4-hydroxy-TEMPO (2,2,6,6-tetramethylpiperidin-1-oxyl) was soluble in saline doped with DMSO but did not show any change in *T*_1_ relaxation times at 10 minutes, suggesting it underwent rapid bioreduction and lost its paramagnetism ruling it out as a reference.

## 4. Conclusions

As a conclusion, we have developed a fully organic, stable, and water-soluble compound with the ability to enhance relaxation in MRI. The compound displayed similar behavior to an existing MRI contrast agent, Gd(DTPA), concerning accumulation and retention in the tumor area. Unfortunately, the targeting effects could not be confirmed with this study. Also, due to the low relaxivity of the compound, it is an unlikely candidate to replace the existing contrast agents as such. However, this compound could be expected to bring an addition to the established MRI diagnostics by opening new ways to study the growing group of metal-free contrast agents for MRI.

## Figures and Tables

**Table 1:** Apparent tumor CA concentration in each animal.

	Animal	Injected dose (*μ*mol/g)	Injected dose (*μ*mol)	Apparent tumor concentration (mM∼*μ*mol/g)	%ID/g
TEEPO-Glc	1	2.2	693	0.67	0.10
	2	1.6	615	0.85	0.14
	3	1.2	385	0.83	0.22
	4	1.2	285	0.59	0.21
	5	1.0	206	0.65	0.32
	6	1.0	210	0.48	0.23
	7	0.5	105	0.09	0.09

Average					0.19 ± 0.08

Gd(DTPA)	3	0.1	34	0.12	0.37
	8	0.1	37	0.05	0.15
	9	0.1	37	0.09	0.24
	10	0.1	33	0.09	0.27

Average					0.25 ± 0.09

## Data Availability

The graphical data used to support the findings of this study are included within the article. The numerical data used to form the graphs are available from the corresponding author upon request.
